# Human endoglin-CD3 bispecific T cell engager antibody induces anti-tumor effect *in vivo*

**DOI:** 10.7150/thno.53121

**Published:** 2021-04-19

**Authors:** Liping Zhong, Wei Shi, Lu Gan, Xiuli Liu, Yu Huo, Pan Wu, Zhikun Zhang, Tao Wu, Hongmei Peng, Yong Huang, Yongxiang Zhao, Yulin Yuan, Zhiming Deng, Hongliang Tang

**Affiliations:** 1 National Center for International Research of Bio-targeting Theranostics, Guangxi Key Laboratory of Bio-targeting Theranostics, Guangxi Medical University, Nanning, Guangxi 530021, China. Collaborative Innovation Center for Targeting Tumor Diagnosis and Therapy, Guangxi Medical University, Nanning, Guangxi 530021, China.; 2 The First People's Hospital of Changde City, Changde, Hunan 41500, China; 3 Department of Oncology, The First Affiliated Hospital, Guangxi University of Chinese Medicine, Nanning, Guangxi 530023, China.; 4 Department of Laboratory Medicine, The People's Hospital of Guangxi Zhuang Autonomous Region, Nanning, Guangxi 530021, China.; 5 Department of Scientific Research, The Affiliated Fangchenggang Hospital, Guangxi University of Chinese Medicine, Fangchenggang, Guangxi 538001, China.

**Keywords:** endoglin, bispecific T-cell engager antibody, neoangiogenesis, immune therapy.

## Abstract

**Rationale:** Endoglin, also known as CD105, is a homo-dimeric membrane glycoprotein required for angiogenesis and serves as a marker for cancer vasculature. In this study, we constructed a bispecific T-cell engager (BiTE) antibody that targets human endoglin and CD3 (hEND-CD3/BiTE). We examined BiTE binding to endoglin-expressing cells and its effects on the cytolytic activity of T cells and cancer development.

**Methods:** The *in vitro* effects of hEND-CD3/BiTE, including binding to target cells, T-cell activation, proliferation, and cytotoxicity, were examined in endoglin-expressing 293T cells, human umbilical vascular endothelial cells, tumor-derived endothelial cells, and CD3^+^ T cells. An *in vivo* xenograft tumor model was established using A549 human lung cancer cells. The therapeutic efficacy of hEND-CD3/BiTE was assessed by monitoring tumor growth, angiogenesis, and mouse survival.

**Results:** hEND-CD3/BiTE specifically bound to endoglin-expressing cells and CD3^+^ T cells *in vitro* and stimulated T-cell activation, proliferation, and Th1 cytokine secretion, and promoted T-cell-mediated cytolysis of endoglin-expressing cells. The hEND-CD3/BiTE *in vivo* caused minimal toxicity to major organs, reduced tumor neoangiogenesis, inhibited tumor growth, and significantly improved mouse survival.

**Conclusions:** Our study demonstrated the therapeutic potential of hEND-CD3/BiTE and provided a novel approach to clinical cancer treatment.

## Introduction

Surgery, chemotherapy, and radiation therapy used either alone or in combination remain the primary approaches in cancer treatment. Chemotherapy and radiotherapy can target recurrences and metastases that cannot be addressed using surgical excision; however, they lack the specificity to distinguish between normal and cancerous tissues, and are frequently associated with severe systemic toxicities and bystander effects on normal cells, and induce preferential growth of drug- or radiation-resistant tumor cells 1-3. In contrast, immunotherapy has long been proposed as an ideal anti-cancer approach with promising efficacy and tolerability, by activating cancer-specific immunity for therapeutic efficacy 4, 5. Immunotherapy includes passive approaches in which anti-cancer monoclonal antibodies or T cells are transferred to bypass activation of endogenous immunity, and directly fight cancer 6, and induce *de novo* cancer-specific immunity 7. Antibodies targeting non-immunomodulatory cancer-related antigens (passive immunotherapy) have been well established for decades, including those involved in the growth or death of tumor cells and non-immune stromal cells, such as vascular endothelial cells and fibroblasts. However, recent clinical studies strongly supported the efficacy of active immunotherapy by antibodies targeting immune checkpoints. These included cytotoxic T-lymphocyte-associated antigen 4 (CTLA-4), programmed cell death protein 1 (PD-1), and chimeric antigen receptor therapy (CAR), resulting in significant cancer remission and survival benefits 8, 9. The ultimate goal of cancer immunotherapy is to activate tumor-specific cytotoxic T lymphocytes (CTLs) and eradicate tumor cells.

Tumor cells develop multiple mechanisms to evade T cell surveillance during cancer development, resulting in deficient recognition of tumors by T cells, acquired resistance to T-cell-mediated killing, induction of T-cell anergy and apoptosis, and accumulation of immunosuppressive Tregs 10. An ideal therapeutic strategy would, therefore, specifically enhance recognition of tumor cells by T cells and stimulate activation/expansion of CTLs. In this regard, bispecific T-cell engager (BiTE) antibody provides an attractive solution. BiTE is an artificial bispecific monoclonal antibody consisting of two single-chain variable fragments (scFv), one of which binds to T cells through the CD3 receptor and the other to a tumor-specific antigen. By linking T cells with tumor cells, BiTE recruits and activates T cell cytotoxicity to tumor sites in the absence of MHC-I or co-stimulatory molecules 11-13. Blinatumomab, a CD19/CD3 BiTE, was the first BiTE antibody approved by the FDA in the clinic for refractory acute lymphoid leukemia treatment 14. Several other BiTEs are currently in clinical trials for various human cancer types, all targeting tumor-specific antigens, including epithelial cell adhesion molecule (EpCAM), carcinoembryonic antigen, CD123, and CD20 15.

Angiogenesis plays an essential role in supporting continuous tumor growth and metastasis, the latter accounting for more than 90% of cancer-related deaths. Targeting angiogenesis is thus a promising therapy and has been approved in cancer treatment. Most angiogenesis inhibitors in the clinic target vascular endothelial growth factors (VEGFs) or their receptors 16. In contrast to tumor cells, which are highly heterogeneous and susceptible to mutations in response to microenvironmental alterations, chemotherapy or radiotherapy, vascular endothelial cells are genetically stable throughout the progression of most solid tumors, readily accessible to therapeutic agents, and less likely to develop resistance to anti-angiogenic therapy 17. Furthermore, tumor vascular endothelial cells present differing phenotypes compared with normal vascular endothelial cells, enabling specific targeting of tumor vasculature 18, 19. Intensive studies have been devoted to identifying and characterizing key biomarkers for tumor angiogenesis. Endoglin, also known as CD105, is a homo-dimeric cell membrane glycoprotein and co-receptor for transforming growth factor β (TGF-β) 20. It is highly expressed on proliferating vascular endothelial cells, specifically tumor-associated vascular and lymphatic endothelium, and in response to hypoxia and inhibition of VEGF signaling 21-23. These features make endoglin a critical marker for tumor angiogenesis and an ideal target for anti-angiogenic treatment, especially in combination with VEGF inhibitors 24. In 2004, Korn et al. first constructed scDb EDGCD3 against endoglin, activating T cells to target killing of endoglin^+^ cells *in vitro*, but the effect was not studied *in vivo* 25.

In this study, we designed and generated a BiTE targeting human endoglin and CD3 (hEND-CD3/BiTE) and characterized the *in vitro* features, including binding to target cells and promoting T-cell activation, proliferation and cytolysis. We also examined *in vivo* biological activities of hEND-CD3/BiTE on cancer progression in a xenograft mouse model of lung cancer. Our goal was to extend the current BiTE strategy (linking T cells with tumor cells) to link T cells with other stromal cells and explore the combination immunotherapy potential with anti-angiogenic cancer treatments.

## Materials and Methods

### Reagents

The cloning/expression plasmid pET-28a (+) was purchased from Invitrogen (Carlsbad, CA, USA). The following antibodies were used in this study: PerCP-conjugated anti-His-tag (ab117496), anti-endoglin (ab230925), and anti-CD34 (ab187282; Abcam, Cambridge, MA, USA); PE-conjugated anti-endoglin (12-1057), FITC-conjugated anti-CD4 (11-0048), PE-conjugated anti-CD8 (15-0088), PerCP-Cyanine5.5-conjugated anti-CD8a (45-0088-41), PE-conjugated anti-CD69 (12-0699), and PE-conjugated anti-CD25 (12-0259; eBioscience, San Diego, CA, USA); in addition to OKT3 (human CD3 monoclonal antibody; NDC: 59676-101) (Ortho Clinical Diagnostics, Raritan, NJ, USA); and anti-proliferating cell nuclear antigen (PCNA) (BM0104; Boster, Pleasanton, CA, USA). Recombinant human endoglin (1097-EN-25) and recombinant human interleukin-2 (IL-2; 202-IL-050) were purchased from R&D (Minneapolis, MN, USA). The fluorescent cell-staining dye carboxyfluorescein succinimidyl ester (CFSE; 00-6993) was from eBioscience, and the PKH26 Red Fluorescent Linker Kit was from Sigma (St. Louis, MO, USA).

### Cells and experimental animals

The *E. coli* strain BL21 (DE3) was purchased from GenScript (Nanjing, China), and competent cells were prepared in our laboratory using the electro-transformation method.

The human lung adenocarcinoma cells A549 (v-054279), human umbilical vascular endothelial cells HUVEC (CRL-1730) and human embryonic kidney 293T cells were purchased from the American Type Culture Collection (ATCC; Manassas, VA, USA) and cultured according to the ATCC instructions. Tumor-derived endothelial cells (Td-EC) were isolated from mouse A549 xenograft tumors using CD105 Microbeads (130-051-201, Miltenyi Biotech, San Diego, CA, USA) following the manufacturer's instructions. Human endoglin-expressing 293T cells (293T-hE) were purchased from GenScript and cultured according to the manufacturer's instructions. The peripheral blood mononuclear cells (PBMCs) were isolated from peripheral blood samples of healthy donors by density centrifugation on Ficoll gradient, as previously described 26. Experiments involving human samples were approved by the Institutional Review Board of Guangxi Medical University (Guangxi, China).

Female specific antigen-free (SPF) immune-deficient, non-obese diabetic/severe combined immunodeficiency (NOD/SCID) mice (age 4-6 weeks) deficient in both B and T cells were purchased from Vital River (Beijing China) and maintained in the SPF experimental animal facility (Guangxi Medical University). All animal experiments were approved by the Institutional Animal Care and Use Committee (IACUC) of the University.

### Construction, expression, purification, and characterization of hEND-CD3/BiTE and control hENDM-CD3/BiTE

We synthesized the coding sequences for V_H_ and V_L_ regions of human endoglin and human CD3 antibodies based on previously published sequences 27, 28. We then used Primer 5.0 software (Primer Biosoft, Palo Alto, CA, USA) to design primers for the V_H_ and V_L_ regions of these two antibodies, and introduced the 15-amino-acid (GGGGS)_3_ linker sequence between the endoglin ScFv and CD3 ScFv fragments. Following the subsequent overlap replication-competent retrovirus (RCR), the linker-containing the hEND-CD3 PCR product was synthesized, an *EcoRI* restriction site was introduced into the upstream region of the endoglin V_H_ sequence, and an *XhoI* restriction site was introduced into the downstream region of the CD3 V_L_ sequence. The PCR product was cleaved with the restriction enzymes *EcoRI* and *XhoI,* cloned into the T7-promoter-containing PET-28a (+) plasmid (which added a His-tag to the N-terminal of the endoglin V_H_ fragment), and was transformed into *E. coli* BL21(DE3) competent cells. *E. coli* cells containing the recombinant plasmids were inoculated into 5 mL of LB broth containing 100 µg/mL kanamycin and rocked at 200 rotations/min overnight at 30 ºC. The overnight cultures were then transferred at a 1:50 ratio to 50 mL of fresh LB medium and were grown at 30 ºC until an OD value at 600 nm of 0.6 to 0.8 was reached.

Isopropyl-β-D-thiogalactopyranoside (IPTG) was added to a final concentration of 0.2 mM, and cultures were grown for a further 4 h at 37 ºC. Cells were then harvested by centrifugation and re-suspended in 10 mM Tris-HCl pH 8.0. Subsequently, cells were sonicated on ice at 200 watts (4 s on and 6 s off cycles) for a total of 15 min and centrifuged at 13,000 rpm/min for 10 min at 4 ºC. After three washes with cold PBS, the inclusion body was dissolved using LE buffer at room temperature for 45 min. The supernatant was collected, filtered through a 0.45-μm strainer and purified using the AKTA pure chromatography system (GE Healthcare, Pittsburgh, PA, USA). The renaturation of the inclusion body was achieved through dialysis in a urea series with decreasing concentrations from 8 M to 6, 4, 2, 1, 0.5 and 0 M. As a control for hEND-CD3/BiTE, we introduced point mutations into the V_H_ region of the endoglin antibody that disrupted the interaction with endoglin, referred to as hENDM-CD3/BiTE.

### Flow cytometry-based *in vitro* assays

To examine the *in vitro* binding of hEND-CD3/BiTE to 293T, 293T-hE, HUVEC, Td-EC or PBMCs, target cells were re-suspended in RPMI1640 medium containing 10% fetal calf serum at 5×10^6^-1×10^7^/mL. For 293T, 293T-hE, HUVEC and Td-EC cells, 100 μL of the cell suspension was incubated with hEND-CD3/BiTE or hENDM/CD3-BiTE (final concentration 10 μg/mL) at 4 ºC for 30 min. The cells were then washed twice with PBS. For the detection of BiTE, a PerCP-conjugated anti-His tag antibody was used. Flow cytometry was performed with Epics XL (Beckman Coulter, Brea, CA, USA). For PBMCs, 100 μL of the cell suspension was incubated with hEND-CD3/BiTE or hENDM/CD3-BiTE at 4 ºC for 30 min. The cells were washed twice with PBS and further incubated with either FITC-conjugated anti-CD4^+^ PerCP-conjugated anti-His tag antibody or PE-conjugated anti-CD8^+^ PerCP-conjugated anti-His tag antibody at 4 ºC for 30 min. For co-targeting of both endothelial cells and T cells, endothelial cells (1×10^5^) were stained with red fluorescent PKH26 dye according to the manufacturer's instructions and incubated with hEND-CD3/BiTE (final concentration 10 μg/mL) at 4 ºC for 2 h. After washing with PBS three times, 2×10^6^ CFSE-labeled PBMCs (as previously described 29) were added to endothelial cells and incubated at 37 ºC for 2-8 h. All cells were imaged under an inverted fluorescence microscope (Eclipse 80i, Nikon, Tokyo, Japan).

To examine T-cell activation, following the co-culture of PBMCs with 293T or 293T-hE cells in the presence of hEND-CD3/BiTE (final concentration 10 µg/mL) at 37 ºC for 48 h, the cells were incubated with FITC-conjugated anti-CD4, PerCP-Cyanine5.5-conjugated anti-CD8α, and PE-conjugated anti-CD69 or PE-conjugated anti-CD25 at 4 ºC for 30 min and analyzed by flow cytometry.

To analyze T-cell proliferation, BSA, hEND-CD3/BiTE, endoglin, or OKT3 (final concentration 10 μg/mL) were used to coat the 96-well plate before the CFSE-labeled PBMCs were seeded into the plate (1×10^6^/well, triplicate for each condition). After incubation at 37 ºC for 72 h, the cells were examined using flow cytometry and analyzed with ModFit LT software (Verity Software, Topsham, ME, USA).

To measure T-cell-mediated cytolysis, PKH26-labeled cells (target cells) were incubated with freshly isolated PBMCs (effector cells) at E:T ratios of 0:1, 2.5:1, 5:1, 10:1, and 20:1. hEND/CD3-BiTE was added into the co-culture at 0, 10^-3^, 10^-2^, 10^-1^, 1, 10 and 100 μg/mL. Cells were co-cultured at 37 ºC for 18 h. The cells were incubated with propidium iodide, and PKH26^+^PI^+^ cells were detected by flow cytometry as apoptotic endothelial cells. The cytolysis percentage was calculated as follows: (PKH26^+^PI^+^% in the experimental group- PKH26^+^PI^+^% in the target cells alone group)/ (1-PKH26^+^PI^+^% in the target cells alone group) ×100%.

### Surface plasmon resonance analysis

hEND-CD3/BiTE was dissolved in HBS-EP solution (0.01M HEPES, pH7.4, 0.15 M NaCl, 3 mM EDTA, and 0.005% P20) at different concentrations and injected continuously onto carboxymethylated dextran 5 (CM5) sensor chips (GE Healthcare, Pittsburgh, PA, USA) coated with antigen CD3ε or CD105. An antigen-free chip was used as a reference. The binding properties of BiTE were measured using Biacore T2000 (GE Healthcare).

### Enzyme-linked immunosorbent assay (ELISA) for cytokine secretion

To examine the cytokine production of T cells in response to BiTE treatment, PBMCs (effector cells) were seeded into 96-well plates together with 293T or 293T-hE cells (target cells) at an E: T ratio of 20:1 (in triplicate for each condition). hEND-CD3/BiTE was then added into the above at a final concentration of 10 μg/mL. The supernatant was collected after 72 h incubation at 37 ºC, and the secretion of IL-10, interferon-γ (IFN-γ), and tumor necrosis factor α (TNF-α) were measured using ELISA kits for these cytokines (Neobioscience, Shenzhen, China).

### *In vivo* xenograft tumor model

To establish a xenograft tumor model, A549 cells (3×10^6^ in 100 μL PBS; target cells) were mixed with freshly isolated PBMCs (6×10^7^ in 100 μL PBS; effector cells) at the E:T ratio of 20:1 and subcutaneously injected into the right axilla of the nude mice (Day 0). From Day 0 to Day 4, hEND-CD3/BiTE in 100 μL PBS was injected daily into the tail vein at 1, 10, or 100 μg. hENDM-CD3/BiTE; PBS was used as a control. To assess the *in vivo* specificity of hEND-CD3/BiTE, recombinant human endoglin was added into hEND-CD3/BiTE solution to a final concentration of 100 μg/mL prior to injection into the tail vein. The tumor length (L) and width (W) were measured every five days starting from Day 5 until Day 30, and the tumor volume (V) was calculated as V = L × W^2^/2.

To evaluate its *in vivo* safety, hEND-CD3/BiTE was intravenously injected into the mice receiving A549 and PBMCs at 20 mg/mL PBS daily for five consecutive days. Age-matched mice not receiving any injection or treatment were used as controls.

### Immunohistochemistry (IHC) analysis

All mice were sacrificed on day 30 after A549 inoculation. To assess the *in vivo* safety of hEND-CD3/BiTE, the liver, heart, spleen, lung, and kidney were isolated from mice receiving 20 mg/mL of BiTE for five days, followed by their embedment in paraffin and staining with hematoxylin and eosin (HE).

For mice bearing xenograft tumors and receiving hEND-CD3/BiTE treatment, the tumor tissues were isolated, fixed in formalin, and embedded in paraffin. The anti-endoglin and anti-proliferating cell nuclear antigen (PCNA) antibodies were then used for IHC staining of endothelial cells and proliferating cells, respectively. For endoglin staining, slides were imaged at 200× (Eclipse 80i), and four fields containing the highest number of endoglin^+^ vessels from each slide were recorded. The average number of endoglin^+^ vessels for these four fields was defined as the microvascular density (MVD). For PCNA staining, slides were imaged at 400×, and the average number of PCNA^+^ cells from five random fields was recorded.

### Statistical analysis

All statistical analyses were performed using SPSS 15.0 software (IBM, New York, NY, USA). Quantitative data are presented as the means ± SD. A *P* value of < 0.05 was considered statistically significant.

## Results

### Construction and characterization of hEND-CD3/BiTE

**Figure 1:**
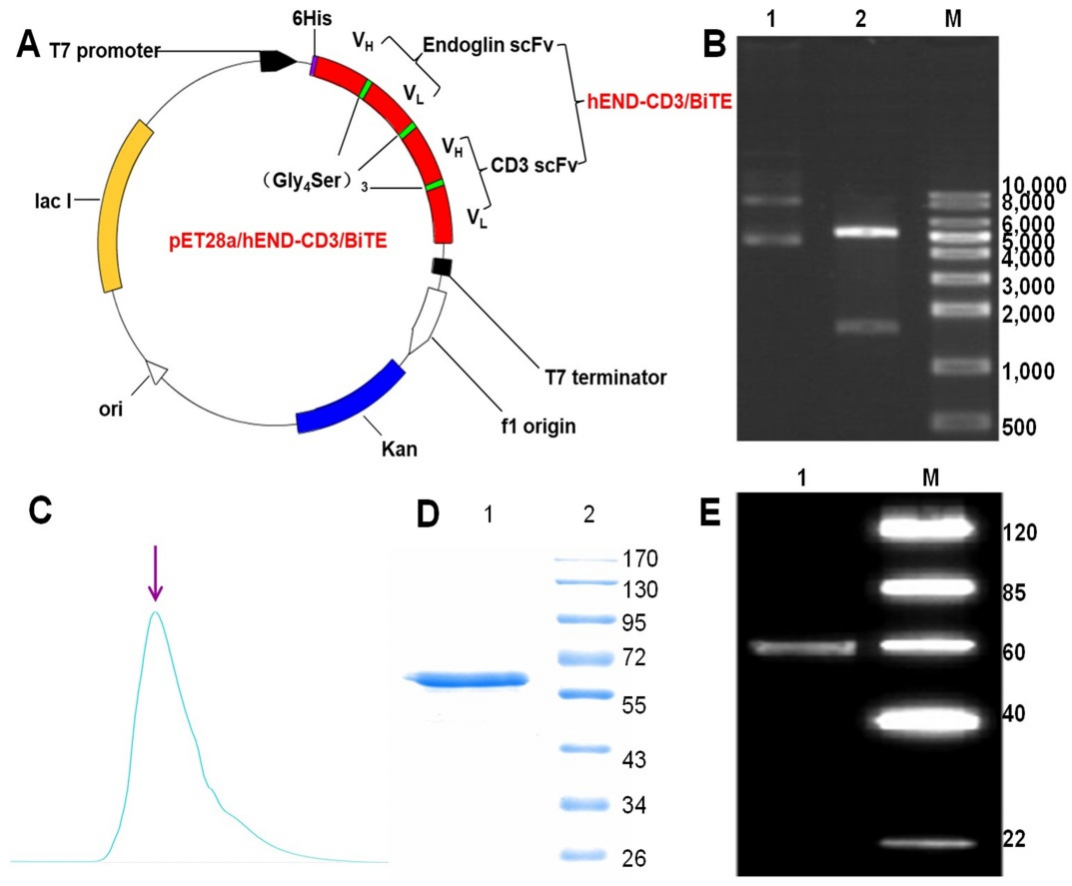
Design, expression, and purification of hEND-CD3/BiTE. (A) The schematic map of expression plasmid pET28a (+) containing human endoglin cDNA linked to human CD3 svFc fragment. (B) Electrophoretogram of parental pET28a (+) plasmid (lane 1) and pET26a (+)-hEND-CD3/BiTE plasmid (lane 2) digested with EcoRI and XhoI. M, size markers. (C) Detection of purified hEND-CD3/BiTE protein eluted from an immobilized metal-affinity chromatography column using a stepwise imidazole gradient. (D) Coomassie blue staining of SDS/PAGE gel of the eluted hEND-CD3/BiTE protein. 1. hEND-CD3/BiTE; 2. Protein molecular weight markers. (E) Western immunoblot analysis of hEND-CD3/BiTE using an anti-His-tag antibody. 1. hEND-CD3/BiTE; M. Protein molecular weight markers.

To generate a bispecific antibody targeting both human endoglin and CD3, we applied overlap PCR, constructed a sequential fusion of VH_endoglin_-VL_endoglin_-VH_CD3_-VL_CD3_ with a 15-amino-acid (GGGGS)_3_ linker sequence between each fragment and His tag at the 5'-end of the endoglin VH fragment. Subsequently, we cloned the fusion gene into the bacterial expression plasmid pET28a (+) (**Figure 1A**). The restriction digestion of the plasmid with *EcoRI* and *XhoI* generated two fragments of 5,369 and 1,641 base pairs (bps) (**Figure 1B**) and sequencing analysis of the 1,641-bp fragment confirmed the identity of the endoglin and CD3 scFv fragments (**Table 1**). Gene expression and protein purification parameters are shown in Supplemental **Figure 1** and Western immunoblot. We acquired a single peptide of approximately 59.5 kDa (**Figure 1C** and **D**), which also reacted with anti-His-tag antibody (**Figure 1E**). The amino acid sequence of hEND-CD3/BiTE is shown in **Table 2**.

### hEND-CD3/BiTE specifically binds to endoglin+ and CD3+ cells

**Figure 2:**
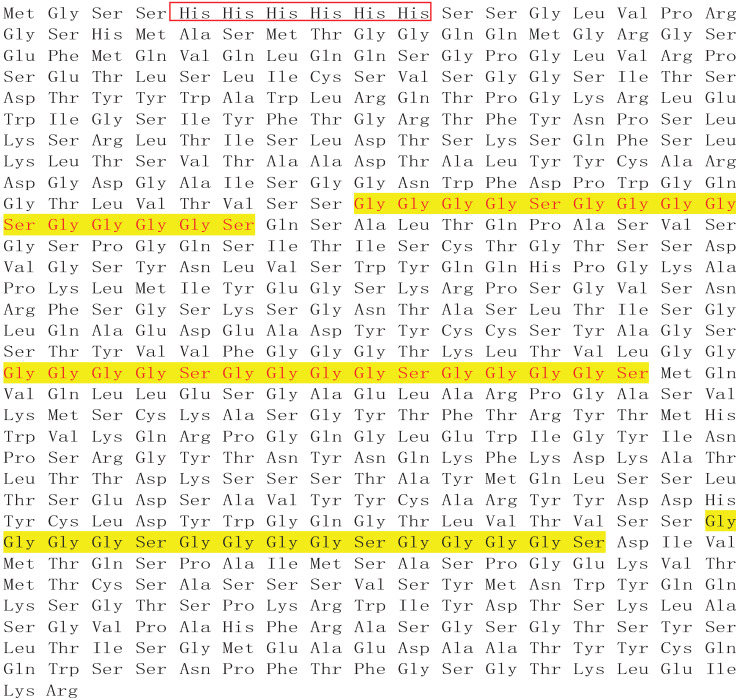

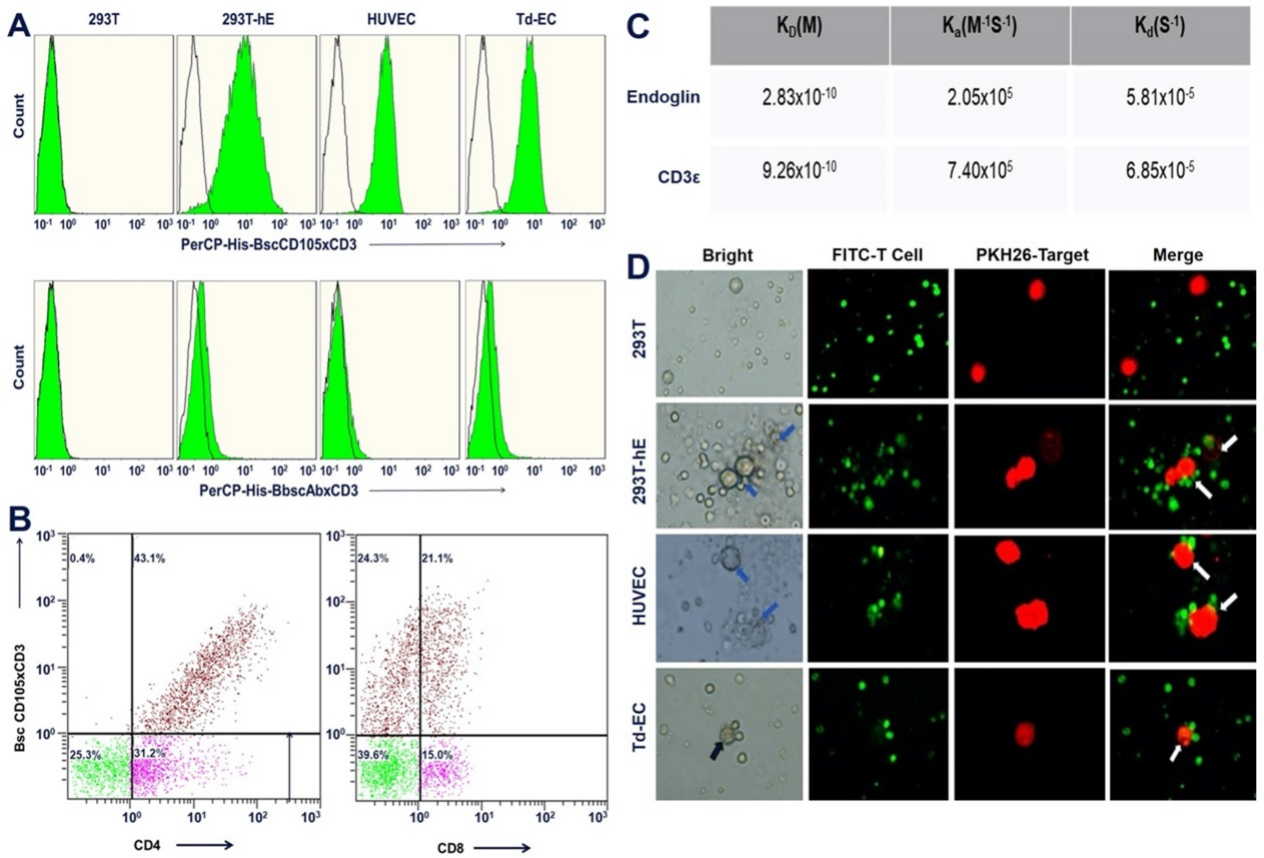
Purified hEND-CD3/BiTE targets endoglin+ cells and CD3^+^ T lymphocytes. (A) hEND-CD3/BiTE (upper panels) or hENDM-CD3/BiTE (lower panels) were incubated with indicated cells, and binding was detected using PerCP-conjugated anti-His-tag antibody (green signal) or isotype-matched control IgG (grey signal) by flow cytometry. (B) hEND-CD3/BiTE was incubated with freshly isolated PBMCs, and its binding among CD4^+^ (left panel) or CD8^+^ lymphocytes was detected using flow cytometry. (C) Binding kinetics and affinity of hEND-CD3/BiTE to the corresponding antigens (endoglin and CD3^+^) were detected by surface plasmon resonance with the association rate constant Ka; dissociation rate constant Kd and equilibrium dissociation constant KD are shown. (D) hEND-CD3/BiTE-mediated linking of FITC-labeled T cells (green) with PKH26-labeled 293T, 293T-hE, HUVEC, or Td-EC was examined by fluorescence microscopy.

We first optimized the concentration gradient of hEND-CD3/BiTE combined with endoglin and PBMCs (as seen in **supplemental Figure 2**). To examine the *in vitro* binding of hEND-CD3/BiTE to endothelial cells, we examined three different endoglin-expressing cells: HUVEC, Td-EC and 293T-hE. We found that hEND-CD3/BiTE bound to all three cell lines with high efficiency (99.8%, 98.7%, and 99.2% for HUVEC, Td-EC, and 293T-hE, respectively); however, there was no binding to non-endoglin-expressing 293T cells (**Figure 2A**).

To evaluate the binding of hEND-CD3/BiTE to T cells, we isolated PBMCs from peripheral blood of healthy donors and detected hEND-CD3/BiTE^+^ cells using anti-His-tag antibody among CD4^+^ or CD8^+^ T cells by flow cytometry. As shown in **Figure 2B**, 43.1% and 21.1% of the CD4^+^ and CD8^+^ T cells, respectively, displayed positive binding to hEND-CD3/BiTE consistent with their relative proportions in total CD3^+^ T cells (**Figure 2B**), suggesting binding of hEND-CD3/BiTE to CD3 molecules on both CD4^+^ and CD8^+^ T cells.

We also used surface plasmon resonance to accurately measure the kinetics of the association rate constant Ka and dissociation rate constant Kd; the affinity was measured by the equilibrium dissociation constant K_D_ of hEND-CD3/BiTE to the antigens, human endoglin and CD3ε. Following the 1:1 Langmuir kinetic model, the affinities of hEND/CD3-BiTE to both endoglin and CD3ε were of the same high magnitude, with binding to the former (K_D_=2.83×10^-10^) somewhat higher than the latter (K_D_=9.26×10^-10^) (**Figure 2C**).

By labeling the endoglin^+^ cells (HUVEC, Td-EC, and 293T-hE) with the red fluorescent probe PKH26 and T cells with green fluorescent CFSE, we showed that hEND-CD3/BiTE efficiently established direct contacts between T cells and endothelial cells but not between non-endoglin-expressing 293T and T cells (**Figure 2D**).

### hEND-CD3/BiTE stimulates T-cell activation, proliferation, and Th1 cytokine production

**Figure 3:**
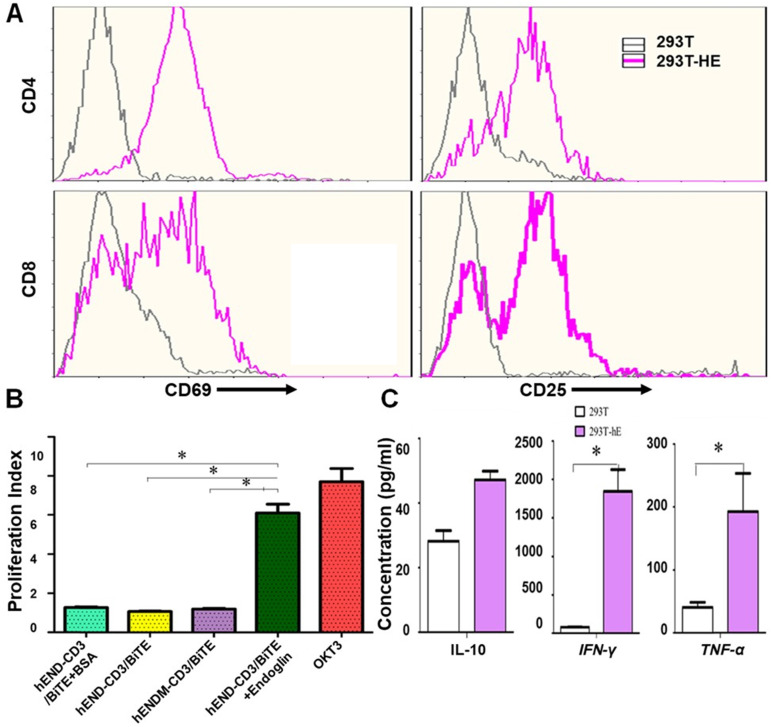
hEND-CD3/BiTE activates T cells, stimulates T-cell proliferation, and increases secretion of Th1 cytokines. (A) PBMCs were co-cultured with 293T (grey signal) or 293T-hE (pink signal) cells in the presence of hEND-CD3/BiTE. The expression of T-cell activation markers CD69 (left panels) and CD25 (right panels) was detected in CD4^+^ (upper rows) and CD8^+^ (lower rows) by flow cytometry. (B) Proliferation of CFSE-labeled T cells in response to hEND-CD3/BiTE and other indicated reagents in the solid phase was measured using flow cytometry. The proliferation index was calculated using the ModFit LT software. (C) Supernatants were collected from the co-cultures of PBMCs with 293T (white bar) or 293T-hE cells (purple bar) in the presence of hEND-CD3/BiTE. The secretion of Th1 cytokines, including IFN-γ, and TNF-α, and Th2 cytokine IL10 was measured using ELISA assays. *P < 0.001.

We next examined hEND-CD3/BiTE effects on T-cell functions, including activation, proliferation, and cytokine production. As displayed in **Figure 3A**, hEND-CD3/BiTE in PBMC and 293T co-cultured cells did not activate either CD4^+^ or CD8^+^ T cells, as indicated by the absence of change in CD69 and CD25; however, hEND-CD3/BiTE stimulated the activation of both T-cell subtypes in the co-culture of PBMCs and 293T-hE cells, suggesting the specificity of this bispecific antibody.

By measuring T-cell proliferation, we found that the presence of hEND-CD3/BiTE alone (1.07), hEND-CD3/BiTE+BSA (1.27), or control hENDM- CD3/BiTE+CD105 (1.19) in the solid could not induce T-cell proliferation. Only hEND-CD3/BiTE+CD105 (6.10) or anti-CD3 monoclonal antibody OKT3 (7.69) significantly stimulated T-cell proliferation (**Figure 3B** and **Supplemental Figure 3**).

Consistent with the activation and proliferation of T cells, we showed that pro-inflammatory Th1 cytokine (IFN-γ and TNF-α) secretion was robustly enhanced by hEND-CD3/BiTE acting on PBMC/293T-hE cocultured cells but not on the co-culture of PBMCs with 293T cells (**Figure 3C**).

### hEND-CD3/BiTE promotes T-cell-mediated cytolysis of endoglin+ cells

**Figure 4:**
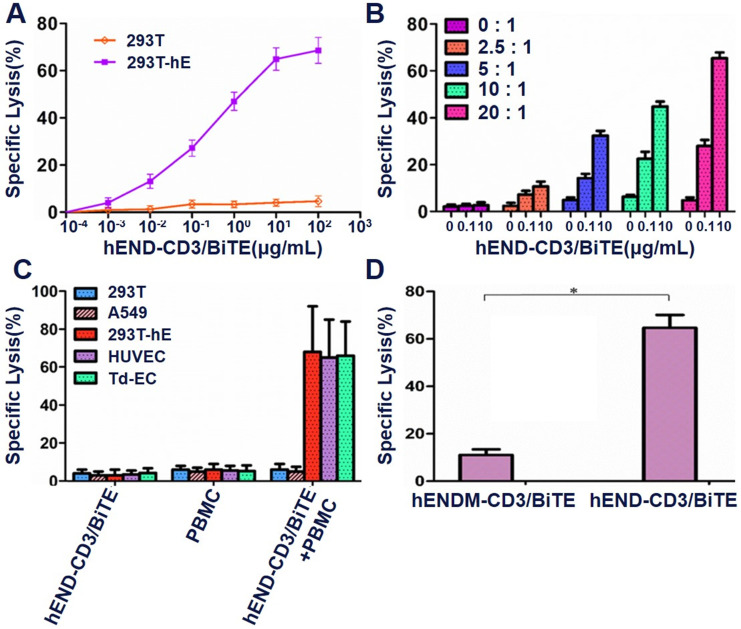
hEND-CD3/BiTE stimulates T-cell-mediated cytolysis of endoglin^+^ cells *in vitro*. PBMCs (effector cells) were co-cultured with indicated target cells labeled with PKH26 in the presence of hEND-CD3/BiTE, and the lysis/apoptosis of target cells was analyzed using flow cytometry and compared with PKH26^+^PI^+^ cells. (A) PBMC-induced cytolysis of 293T (orange line) or 293T-hE cells (purple line) in response to various concentrations of hEND-CD3/BiTE. (B) PBMC-induced cytolysis of 293T-hE cells was assessed at different E:T ratios and hEND-CD3/BiTE concentrations. (C) PBMC-induced cytolysis of 293T (blue bar), 293T-hE (red bar), HUVEC (purple bar), and Td-EC (green bar) at an E:T ratio of 20:1 and/or in response to 10 μg/mL hEND-CD3/BiTE. (D) PBMC-induced cytolysis of 293T-hE cells was compared between hEND-CD3/BiTE and the control hENDM-CD3/BiTE. *P < 0.001.

Increased activation, proliferation, and cytokine production are prerequisite steps for T-cell-mediated cytolysis. By measuring the apoptosis in endoglin^+^ target cells (as represented by PKH26^+^PI^+^ cells by flow cytometry) in the co-culture with PBMCs, we found that the T cells' cytolytic activity increased with hEND-CD3/BiTE concentration (**Supplemental Figure 4**). The T-cell cytolysis presented a near-linear increase with an increased hEND-CD3/BiTE concentration from 10^-4^ to 10 μg/mL, and plateaued after 10 μg/mL (**Figure 4A**). The cytolysis increased with an increased E:T ratio between 0:1 to 20:1 (**Figure 4B**). We, therefore, chose to use 10 μg/mL END/CD3-BiTE and an E:T ratio of 20:1 in future experiments. We found that the cytolysis was specific for endoglin-expressing cells and not 293T cells; it was also effective in 293T-hE, HUVEC, and Td-EC cells (**Figure 4C**) in the presence of hEND-CD3/BiTE but not in the control hENDM-CD3/BiTE (**Figure 4D**).

HEND-CD3/BiTE could induce T cells to kill 293T-HE cells expressing endoglin with a killing index of 66.9%. The killing effect of the negative control antibody hENDM-CD3/BiTE on 293T-HE cells was not obvious with the killing index of 13.59%; the difference was statistically significant. These results suggested that only double-specific single-chain antibodies with antigen-binding sites targeting endoglin could kill endoglin-expressing 293T-HE cells (**Supplemental Figure 5**).

### hEND-CD3/BiTE reduces tumor neoangiogenesis and tumor cell proliferation

**Figure 5:**
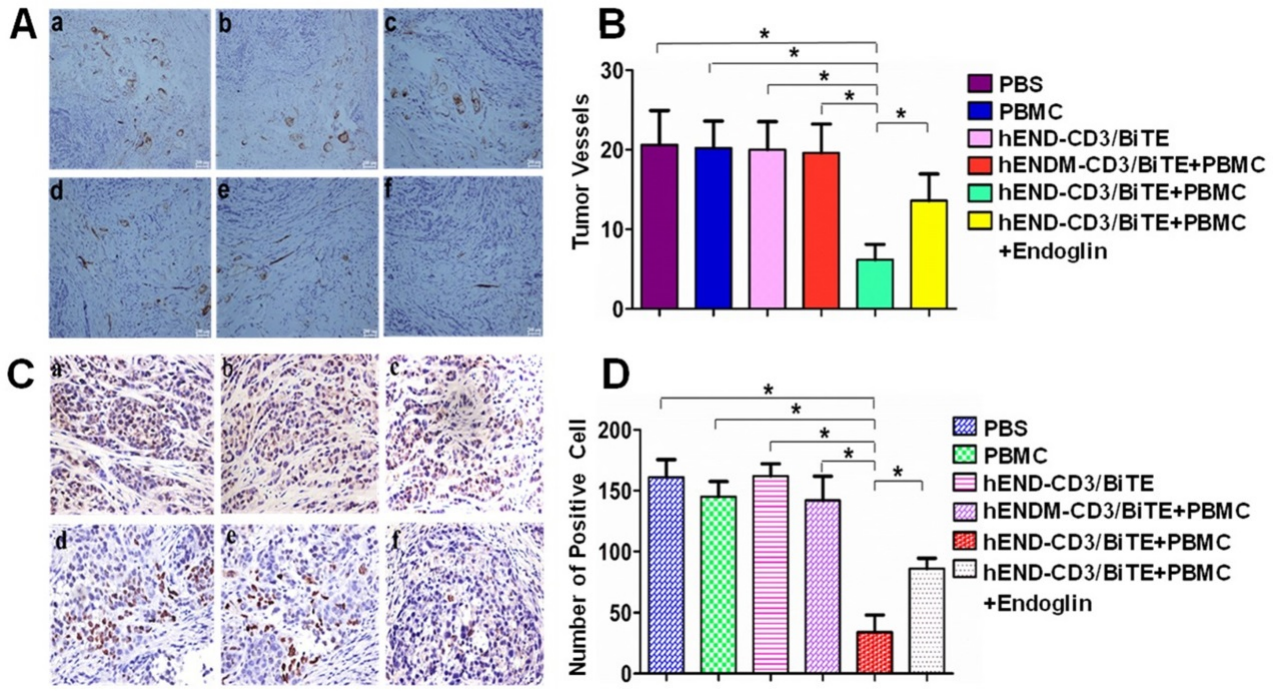
hEND-CD3/BiTE inhibits A549 mouse xenograft tumor angiogenesis and tumor cell proliferation *in vivo*. (A) Vascular endothelial cells within tumors were detected by immunohistochemical staining of endoglin; representative IHC images (200 ×) from each group are presented. (B) Endoglin^+^ vessels in the tumors were quantified and compared between groups. (C) Tumor cell proliferation was examined by immunohistochemical staining of PCNA; representative IHC images (400 ×) from each group are presented. (D) PCNA^+^ cells in the tumors were quantified and compared between groups. a. PBS; b. hEND-CD3/BiTE; c. PBMC; d. hENDM-CD3/BiTE +PBMC; e. hEND-CD3/BiTE +PBMC + Endoglin; f. hEND-CD3/BiTE +PBMC. *P < 0.001.

To assess the *in vivo* cytolytic effect of hEND-CD3/BiTE on tumor vascular endothelial cells, we measured the MVD by immunohistochemical staining of endoglin (**Figure 5**) and CD34 (**Supplemental Figure 6**). As shown in **Figures 5A** and **5B**, co-administration of hEND-CD3/BiTE and PBMCs significantly reduced neoangiogenesis in the tumor (*P*<0.05 compared with all other groups). The addition of recombinant endoglin partially blocked neoangiogenesis inhibition.

Angiogenesis is essential for continued tumor growth. Therefore, we analyzed tumor cell proliferation by PCNA immunohistochemical staining. Consistent with the changes in angiogenesis, co-administration of hEND-CD3/BiTE and PBMCs was associated with a significant reduction in PCNA^+^ proliferating cells (*P*<0.01 compared with all other groups; **Figures 5C** and **5D**).

### hEND-CD3/BiTE inhibits *in vivo* tumor growth and improves mouse survival

We established a xenograft tumor model in nude mice using human lung adenocarcinoma A549 cells with or without human PBMCs from healthy donors and characterized the *in vivo* activity of hEND-CD3/BiTE. We also administered hEND-CD3/BiTE or control hENDM-CD3/BiTE in mice via the tail vein.

**Figure 6:**
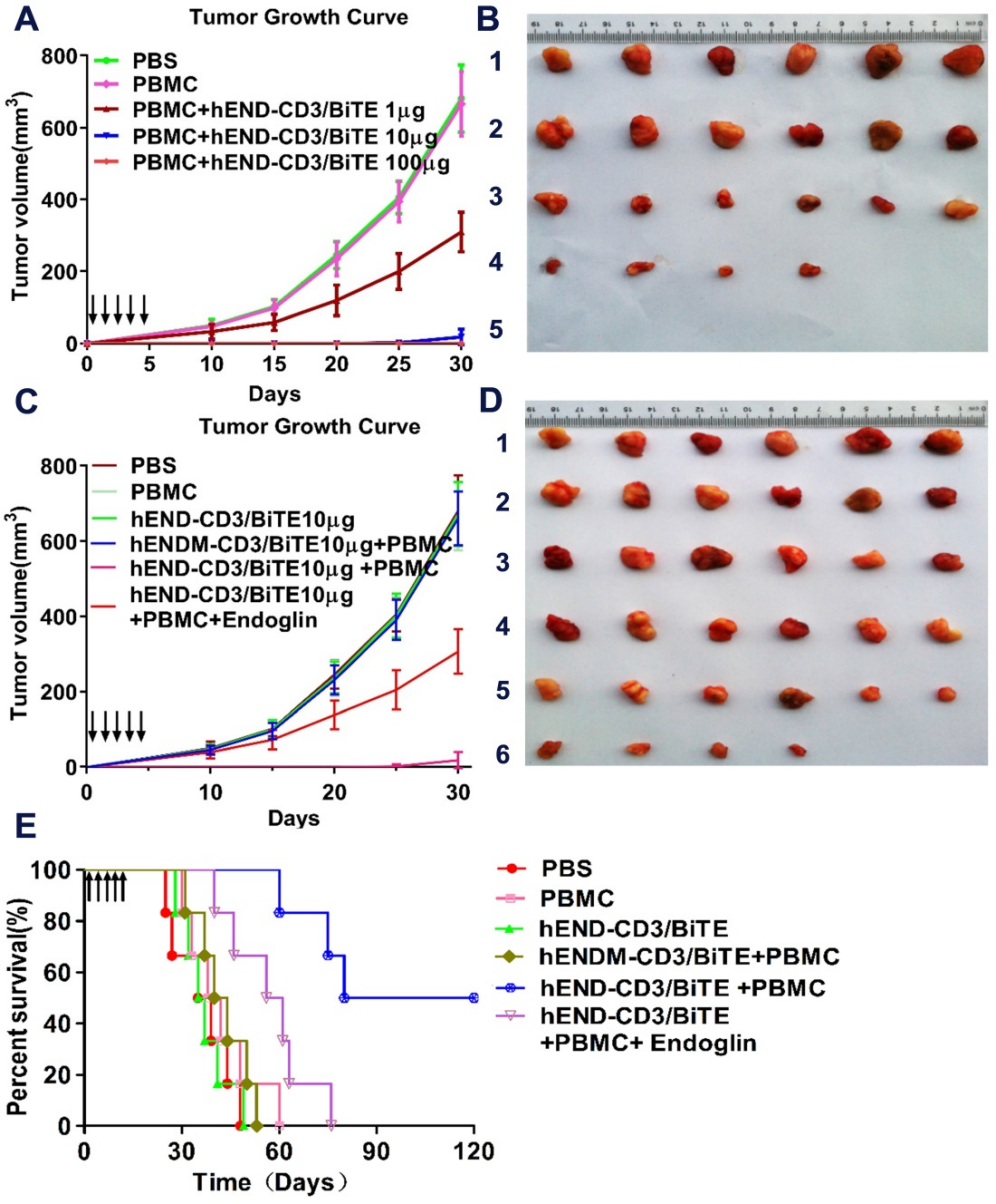
hEND-CD3/BiTE inhibits *in vivo* tumor growth and improves mouse survival. NOD/SCID mice (n = 6) were subcutaneously implanted with A549 cells without (PBS group) or with human PBMCs (PBMC group). Mice were also treated with hEND-CD3/BiTE or control hENDM-CD3/BiTE daily i.v. injections for the first five days. (A) and (B) Dose-response of tumor growth (A) and images of tumors (B) in mice receiving hEND-CD3/BiTE at indicated doses. 1, PBS; 2, PBMC; 3, PBMCs + 1 μg hEND-CD3/BiTE; 4, PBMCs + 10 μg hEND-CD3/BiTE; 5, PBMCs + 100 μg hEND-CD3/BiTE. (C) and (D) Tumor growth (C) and images (D) in the indicated groups of mice. 1, PBS group; 2, PBMC group; 3, hEND-CD3/BiTE group; 4, PBMC+ hENDM-CD3/BiTE; 5, PBMC+ hEND-CD3/BiTE; 6, PBMC+ hEND-CD3/BiTE + endoglin. (E) Kaplan-Meier survival analysis of mice from the indicated groups.

By following tumor growth, we found that the co-injection of PBMCs with A549 without hEND-CD3/BiTE did not significantly affect tumor growth compared with mice receiving A549 cells alone (PBS group). The administration of 1 μg hEND-CD3/BiTE per day for five days significantly inhibited tumor growth. Moreover, treatment with hEND-CD3/BiTE at 10 μg/day for five days led to minimal tumor growth, whereas 100 μg of the antibody completely abolished tumor growth for the first 30 days after A549 injection (**Figure 6A** and **B**).

The tumor inhibitory activity of hEND-CD3/BiTE required the presence of both PBMCs and A549 cells because tumors in mice receiving hEND-CD3/BiTE alone continued to grow at a rate comparable with those in mice receiving only A549 cells (PBS group). The tumor inhibitory effect was also specific for hEND-CD3/BiTE because the control hENDM-CD3/BiTE did not slow tumor growth. Furthermore, co-administration of recombinant human endoglin partially yet significantly reduced tumor growth (**Figure 6C** and **D**).

Consistent with its effect on tumor growth, hEND-CD3/BiTE also significantly improved mouse survival (mean survival time, 100 days; *P* < 0.05) compared with the PBS (mean survival time, 37 d), hEND-CD3/BiTE alone (mean survival time, 36 d), PBMCs alone (mean survival time, 40 d), and hENDM-CD3/ BiTE+PBMCs (mean survival time, 42 d) groups. The addition of recombinant human endoglin to hEND-CD3/BiTE partially extended the mean mouse survival to 58.5 d, which was still significantly shorter than that achieved by hEND-CD3/BiTE, suggesting the blocking effect of recombinant endoglin (**Figure 6E**).

### hEND-CD3/BiTE causes minimal toxicity to major organs

**Figure 7:**
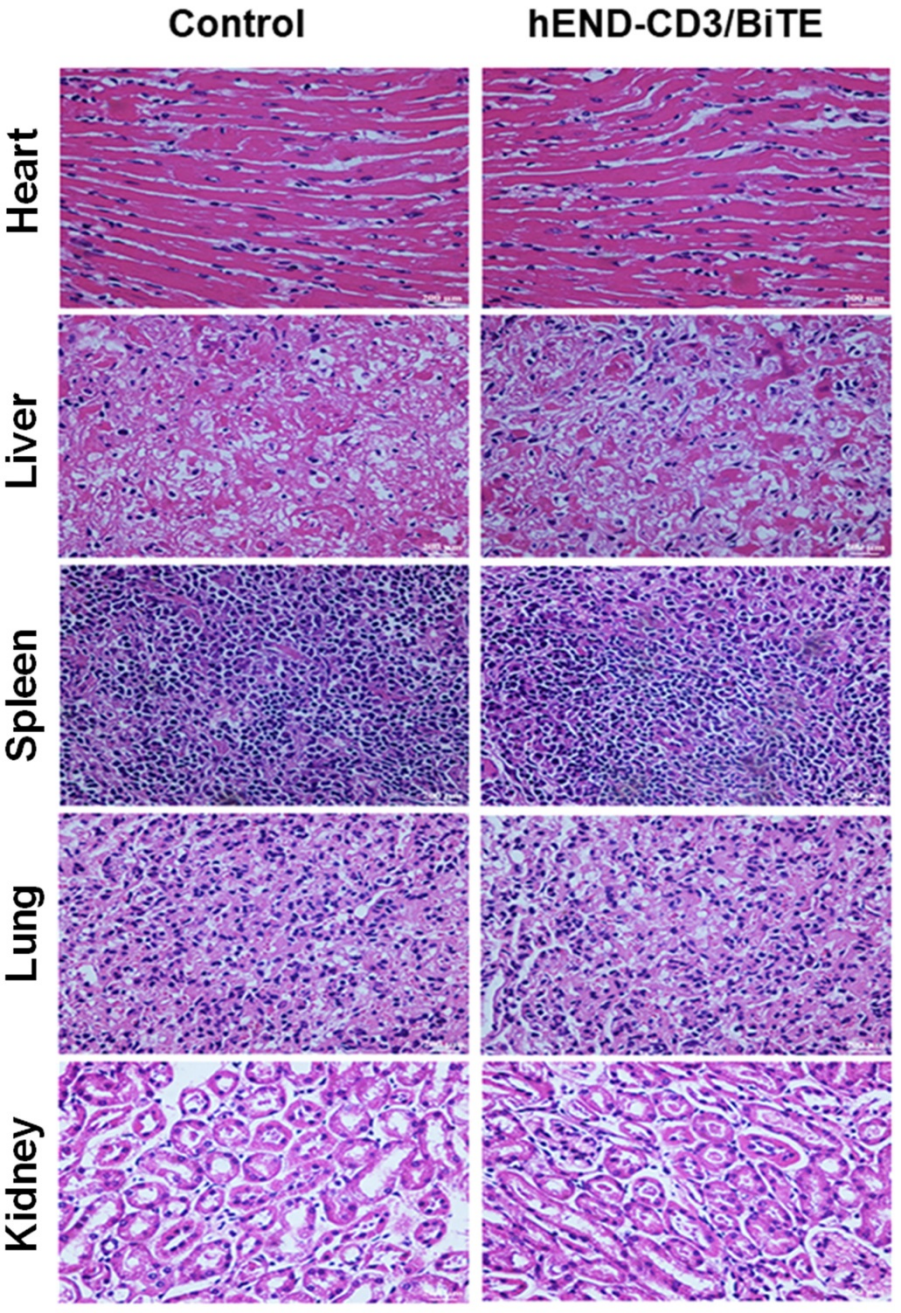
The *in vivo* toxicity of hEND-CD3/BiTE. Mice (n = 6) were subcutaneously implanted with A549 cells and human PBMCs and i.v. injected with 2 mg/day hEND-CD3/BiTE for the first five days. Untreated mice were used as controls. On day 30 after A549 injection, all mice were sacrificed, and the heart, liver, spleen, lung, and kidney isolated, stained with hematoxylin and eosin, and comparisons were made between the two groups; magnification, 400 ×.

Lastly, we assessed the *in vivo* safety of hEND-CD3/BiTE after intravenous injection at 2 mg/day for five days, which was 20-fold higher than the biologically active dose demonstrated above. By analyzing several organs, including the heart, liver, spleen, lung, and kidney, we did not detect tumor metastasis, significant lymphocyte infiltration, or structural alterations in these organs compared with control mice not receiving any injections or treatments (**Figure 7**).

## Discussion

In this study, we generated a bispecific antibody targeting CD3^+^ T cells and endoglin^+^ endothelial cells. We showed that the hEND-CD3/BiTE was not only active in binding to the target cell types but was also biologically significant in activating the T-cell-mediated cytolysis of endothelial cells. These *in vitro* activities also translated to *in vivo* functions by reducing tumor angiogenesis, inhibiting tumor growth, and extending mouse survival.

During tumor progression, angiogenesis provides nutrients to support continuous tumor growth and also creates conduits for the distant dissemination of tumor cells 30. Therefore, targeting angiogenesis becomes a significant strategy in cancer therapy and is under intensive investigation 31. Continued research on the molecular mechanisms controlling tumor angiogenesis has and will continue to reveal important therapeutic targets leading to the development of several approaches, including targeting the key molecules (the most important being VEGFs and their receptors), gene therapy, and neutralizing monoclonal antibodies. Although exogenous genes delivered via a gene therapy strategy have several advantages and show efficacy in various proof-of-principle animal experiments, they must overcome impediments at multiple steps from transcription, translation, post-translational modifications to functional proteins targeting angiogenesis. This approach has not shown clinical efficacy in experimental animals, as demonstrated by *in vitro* studies 32, 33.

Neutralizing monoclonal antibodies, on the other hand, directly target endogenous proangiogenic factors and encounter reduced obstacles. The success of this strategy was first validated by FDA approval of Avastin, a humanized VEGF-neutralizing monoclonal antibody for metastatic colorectal cancer in combination with standard care 34 and subsequently for the treatment of non-small-cell lung cancer and metastatic breast cancer 35, 36. Despite the initial promising performance of neutralizing monoclonal antibodies, especially those targeting VEGF, their clinical benefits are short-term, and patients eventually demonstrate a lack of response or resistance to the anti-angiogenesis therapy. Several mechanisms underlying the resistance phenotype have been proposed and extensively studied 37. One significant mechanism is that tumor cells and other stromal cells may selectively stimulate alternative pro-angiogenic signaling, thereby contributing to anti-VEGF therapy resistance 38-41. Therefore, it is important to identify the mechanisms of alternative pro-angiogenic factors that induce and mediate resistance to anti-VEGF therapy. In this context, the size of the macromolecular antibody may limit its bioavailability and effective concentration in the tumor mass reducing the efficacy of neutralizing antibody 42, 43, which may be addressed by antibody engineering or designing an alternative delivery vehicle. This report proposed a novel strategy to target both mechanisms that may lead to anti-VEGF therapy resistance.

Endoglin, a co-receptor of TGF-β, is highly expressed in tumor-associated vascular and lymphatic endothelium 21, 23, 44. *In vitro* studies demonstrated that anti-tumor therapy targeting endoglin inhibited proliferation, migration, and adhesion and induced apoptosis of endothelial cells by disrupting TGF-β signaling 20, 45, 46. Moreover, endoglin plays a significant role in the development of resistance to VEGF therapy. In mice bearing pancreatic xenografts and treated with anti-VEGF antibody, endoglin was among the few genes that showed significant up-regulation 47. Tumors in mice with one copy of the endoglin gene exhibited a delayed onset of resistance to anti-VEGF treatment 48. Also, high endoglin expression correlated with a worse prognosis in various human solid tumors, including lung, colorectal, prostate, and hepatocellular cancers. 49. Therefore, anti-endoglin therapy combined with VEGF inhibitors would likely provide improved benefits.

Anti-endoglin therapy has consistently demonstrated positive therapeutic outcomes in experimental animal models. Seon *et al.* showed that anti-endoglin monoclonal antibodies (mAbs) inhibited tumor growth and suppressed metastasis by stimulating T-cell immunity resulting from antibody-dependent cell-mediated cytotoxicity and inhibited endothelial growth while promoting tumor cell apoptosis through Fas/FasL signaling 50. Additionally, anti-endoglin mAbs are associated with minimal *in vivo* toxicity, supporting its potential in clinical therapy. Also, in immunocompetent mice, the anti-tumor activity of anti-endoglin mAbs was higher than in immunocompromised SCID mice; the depletion of CD4^+^ and/or CD8^+^ T cells abrogated the anti-tumor efficacy of anti-endoglin mAbs, indicating that T-cell immunity may synergize with the anti-tumor activity of anti-endoglin mAbs 51, 52.

These findings prompted us to develop an approach to boost T-cell immunity and target endoglin simultaneously. Herein, we showed that the bispecific hEND-CD3/BiTE antibody we developed specifically targeted endoglin^+^ cells, including those ectopically expressing endoglin (293T-hE) and normal, as well as tumor endothelial cells expressing endogenous endoglin (HUVEC and Td-EC). This process generated no toxicity and did not induce significant lymphocyte infiltration in the major organs of nude mice even at a dose 20-fold higher than a biologically active dose. However, the further work still needs to study cross-reactions in different species in near future.

BiTE is a bispecific antibody composed of scFv from two monoclonal antibodies, including one targeting CD3. The connection between the two scFv by a short peptide linker enables free rotation of the two arms and enhances the binding affinity to the corresponding antigens. The smaller size due to the minimal binding domains (Fv fragments) of two monoclonal antibodies reduces its antigenicity, thereby increasing its tissue penetration and ensuring the proximity between T cells and target cells. The simple design of BiTE also enables high-yield production for various biomedical applications 53. Most BiTEs developed thus far target tumor-specific antigens in addition to T cells 11, 54. Herein, we utilized BiTE properties to design a bispecific antibody targeting T cells and endoglin. We expected this approach to boost the anti-tumor activity of endoglin inhibitor by activating T-cell immunity and specifically induce the cytolysis of endothelial cells via T-cell immunity.

Following gene expression and protein purification, most proteins were in the inclusion bodies, probably due to their high-level transcription driven by the T7 promoter in BL21(DE3), leading to numerous mis-folded proteins and thus aggregation into inclusion bodies. To renature the proteins, we applied urea with decreasing concentrations from 8 M to 6, 4, 2, 1, 0.5 and 0 M, with each dialysis step lasting for 2 h. We tested the biological activity of the purified and renatured BiTEs by examining their binding to the target cells. The specific binding to endoglin-expressing 293T cells but not to parental 293T cells confirmed its specificity for endoglin. The binding analysis also showed that, among the total PBMCs, 43.1% were positive for CD4 and hEND-CD3/BiTE and 21.1% for CD8 and hEND-CD3/BiTE. The total percentage of these two populations (64.2%) was consistent with the percentage of T cells in PBMCs. Furthermore, their relative ratio reflected the CD4^+^/CD8^+^ cell ratio within the T-cell population, indicating the specific binding of hEND-CD3/BiTE to the two major T cell subpopulations. In the co-culture system containing both PBMCs and endoglin-expressing cells, we directly visualized contacts between the two cell types mediated by hEND-CD3/BiTE but not between PBMCs and non-endoglin-expressing 293T cells.

Further *in vitro* functional studies showed that the hEND-CD3/BiTE could stimulate T-cell activation, proliferation, Th1 cytokine production, and cytolysis; these processes were dependent on the hEND-CD3/BiTE binding to endoglin in a specific cellular context. Consistent with our findings, Choi *et al.* reported that BiTE preferentially stimulated Th1 cytokine production 55. The stimulation of multiple T-cell responses suggested that BiTE activity not only affected CD4^+^ but also CD8^+^ T cells. Future studies should explore the mechanisms underlying specific stimulation of Th1 response by BiTE.

The *in vivo* analysis focused on lung cancer because of its high morbidity and mortality and the significance of angiogenesis and endoglin in lung cancer progression and metastasis 56-58. Consistent with the *in vitro* findings, hEND-CD3/BiTE administration significantly reduced tumor angiogenesis and inhibited tumor cell proliferation, leading to retarded tumor growth and improved mouse survival. In another xenograft model of HepG2-derived hepatocellular carcinoma in mice, we observed potent anti-tumor activity of hEND/CD3/BiTE (data not shown). Furthermore, the *in vivo* effects of hEND-CD3/BiTE were specifically and partially blocked by free recombinant human endoglin, supporting the specificity of this antibody.

It has been reported that in some hematological malignancies, BiTEs, such as blinatumomab, caused the cytokine release syndrome and adverse central nervous system events in a small number of patients 59, 60. No obvious side effects of BiTEs in patients with solid tumors were observed 61, consistent with our results of hEND-CD3/BiTE in solid tumor vessels.

In summary, we proposed a novel design of the BiTE antibody for cancer therapy by targeting CD3^+^ T cells in conjunction with endothelial cells instead of tumor cells. We demonstrated the specificity, efficacy, and safety of this new approach using *in vitro* and *in vivo* analyses. We anticipate that this strategy will lead to promising therapies for various cancers that rely on angiogenesis for continuous growth and metastasis. Specifically, as a combinatorial treatment, this strategy may avoid the development of resistance to anti-VEGF therapy.

## Supplementary Material

Supplementary figures and tables.

## Figures and Tables

**Table 1:**
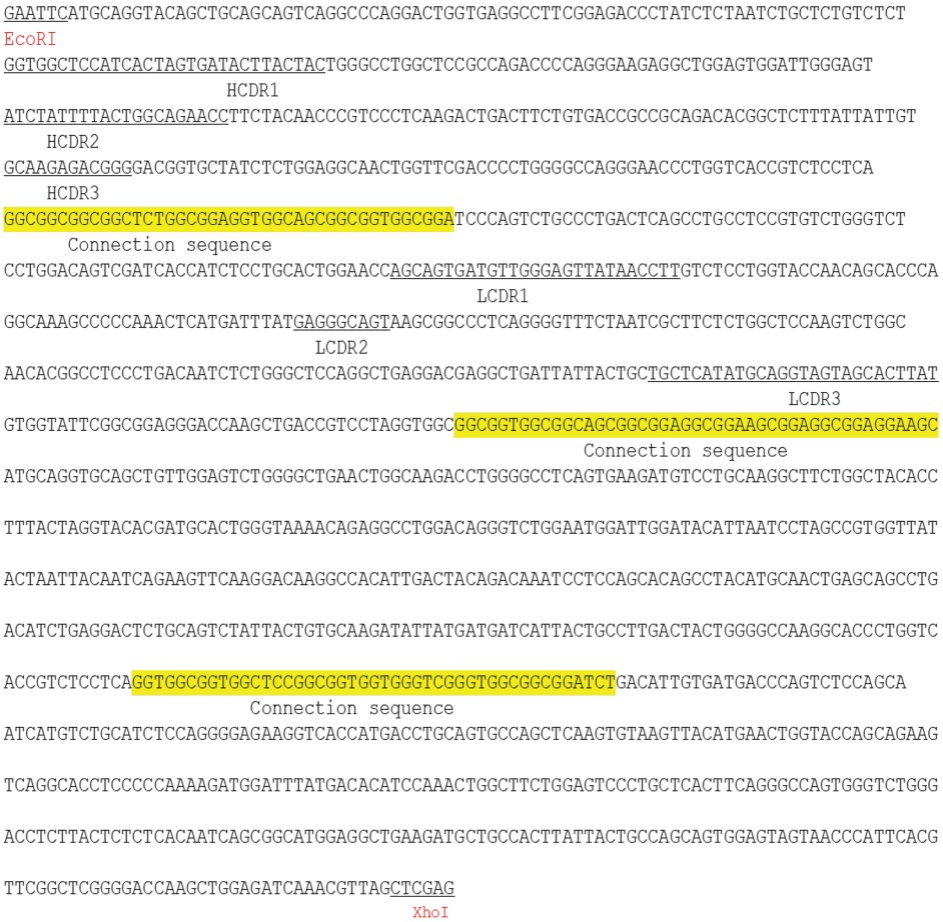
hEND-CD3/BiTE gene sequence.

**Table 2:** hEND-CD3/BiTE amino acid sequence.
