# Human endoglin-CD3 bispecific T cell engager antibody induces anti-tumor effect *in vivo*

**DOI:** 10.7150/thno.117883

**Published:** 2025-08-05

**Authors:** Liping Zhong, Wei Shi, Lu Gan, Xiuli Liu, Yu Huo, Pan Wu, Zhikun Zhang, Tao Wu, Hongmei Peng, Yong Huang, Yongxiang Zhao, Yulin Yuan, Zhiming Deng, Hongliang Tang

**Affiliations:** 1 National Center for International Research of Bio-targeting Theranostics, Guangxi Key Laboratory of Bio-targeting Theranostics, Guangxi Medical University, Nanning, Guangxi 530021, China. Collaborative Innovation Center for Targeting Tumor Diagnosis and Therapy, Guangxi Medical University, Nanning, Guangxi 530021, China.; 2 The First People's Hospital of Changde City, Changde, Hunan 41500, China; 3 Department of Oncology, The First Affiliated Hospital, Guangxi University of Chinese Medicine, Nanning, Guangxi 530023, China.; 4 Department of Laboratory Medicine, The People's Hospital of Guangxi Zhuang Autonomous Region, Nanning, Guangxi 530021, China.; 5 Department of Scientific Research, The Affiliated Fangchenggang Hospital, Guangxi University of Chinese Medicine, Fangchenggang, Guangxi 538001, China.

The authors apologize for errors in the originally published version of this article that require correction. Mistaken images were used in Figure 5A (e), Figure 7 (Heart of the hEND-CD3/BiTE group), and Supplementary Figures S4A, S4C, and S6d. The authors have corrected the mistakenly used images and declare that these corrections do not change the conclusions of this article. The authors sincerely apologize to the journal and its readers for any confusion this may have caused. The corrected versions are provided below.

## Figures and Tables

**Figure A:**
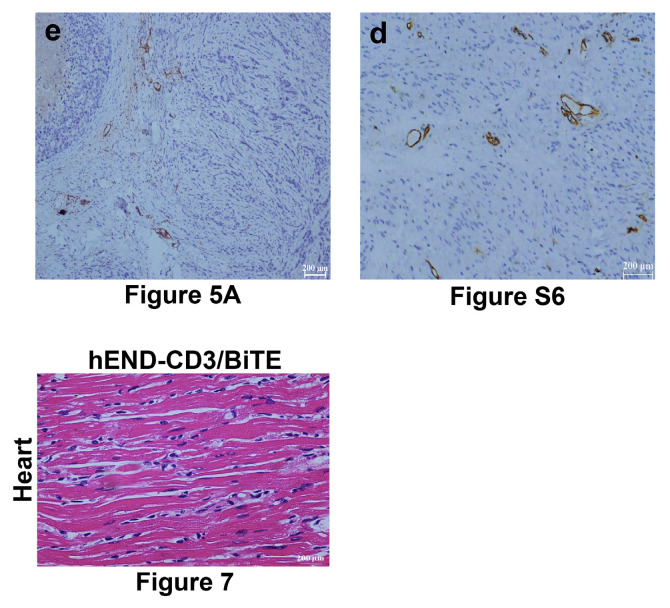
Corrected figure for original Figure 5A (e), Figure 7 (Heart of the hEND-CD3/BiTE group), and Supplementary Figure S6d.

**Figure B:**
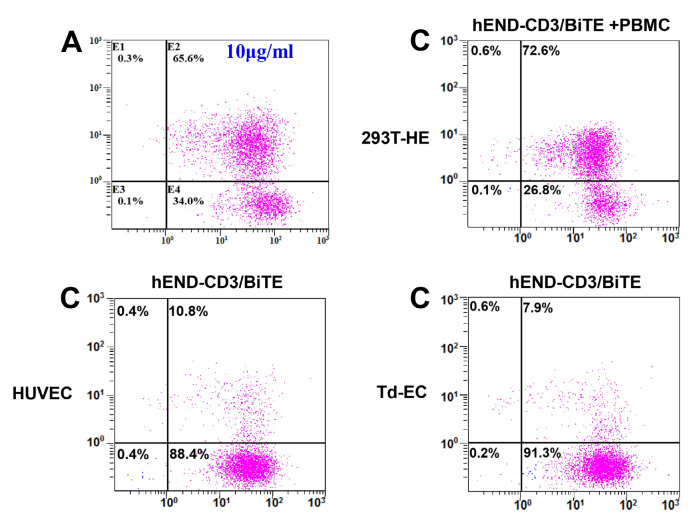
Corrected figure for original Supplementary Figure S4A and C.

