# Supplementary material for: Human endoglin-CD3 bispecific T cell engager antibody induces anti-tumor effect in vivo
Source: Theranostics. 2021 Apr 19;11(13):6393–406. doi: 10.7150/thno.53121 (PMC8120215; doi:10.7150/thno.53121)
Supplement: Supplementary file 1 — Supplementary figures and tables. [file thnov11p6393s1.pdf]

Supplemental Information

Human endoglin-CD3 bispecific T cell engager antibody induces anti-tumor effect *in vivo*

Liping Zhong[1, 2, *], Wei Shi[3, *], Lu Gan[1, *], Xiuli Liu[1, *], Yu Huo[1], Pan Wu[1], Zhikun Zhang[1], Tao Wu[2], Hongmei Peng[2], Yong Huang[1], Yongxiang Zhao[1], Yulin Yuan[1,4, ✉], Zhiming Deng[2, ✉], Hongliang Tang[3, 5, ✉]

[1] National Center for International Research of Bio-targeting Theranostics, Guangxi Key Laboratory of Bio-targeting Theranostics, Guangxi Medical University, Nanning, Guangxi 530021, China. Collaborative Innovation Center for Targeting Tumor Diagnosis and Therapy, Guangxi Medical University, Nanning, Guangxi 530021, China

[2] The First People's Hospital of Changde City, Changde, Hunan 41500, China

[3] Department of Oncology, The First Affiliated Hospital, Guangxi University of Chinese Medicine, Nanning, Guangxi 530023, China

[4] Department of Laboratory Medicine, The People's Hospital of Guangxi Zhuang Autonomous Region, Nanning, Guangxi 530021, China

[5] Department of Scientific Research, The Affiliated Fangchenggang Hospital, Guangxi University of Chinese Medicine, Fangchenggang, Guangxi 538001, China

[*] These authors contributed equally to this work.

✉Correspondence: Hongliang Tang (tanghl204@gxtcmu.edu.cn), Zhiming Deng (dengzhmcd@ 126.com), Yulin Yuan (yuanyulin@126.com)

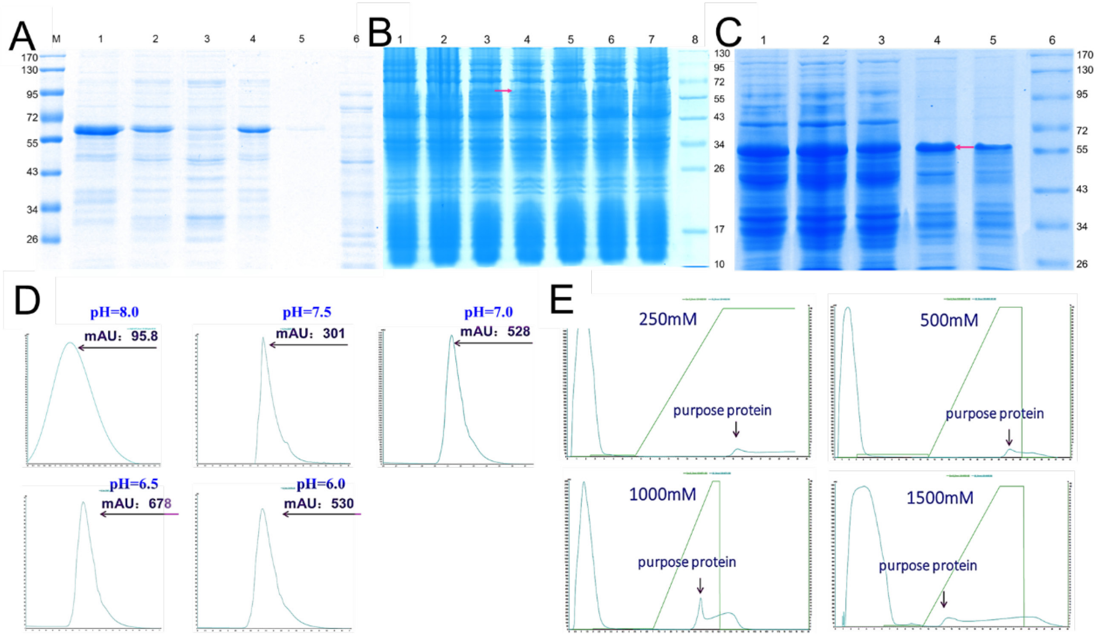

Supplemental Figure 1. Optimized hEND-CD3/BiTE expression and purification conditions. (A) Expression of hEND-CD3/BiTE and its negative control plasmid. M: Marker 1. hEND-CD3/BiTE Precipitate, 2. hEND-CD3/BiTE Supernatant, 3. hENDM-CD3/BiTE Supernatant, 4. hENDM-CD3/BiTE Precipitate, 5. PET28a Supernatant, 6. PET28a Precipitate, (B) IPTG-induced hEND-CD3/BiTE expression gradient. 1. Uninduced, 2. 0.05mmol/L, 3. 0.1mmol/L. 4. 0.2mmol/L, 5. 0.5mmol/L, 6. 1mmol/L, 7. 2mmol/L, 8. Markers. (C) IPTG-induced hEND-CD3/BiTE expression time gradient. 1. 1h, 2. 2h, 3. 3h, 4. 4h, 5. 5h, 6. Markers, (D) Optimized sample buffer pH. At pH 6.5, the elution peak of the target protein was highest. (E) Optimized eluent imidazole concentration. At 1000 mM imidazole, the elution target protein peak was highest.

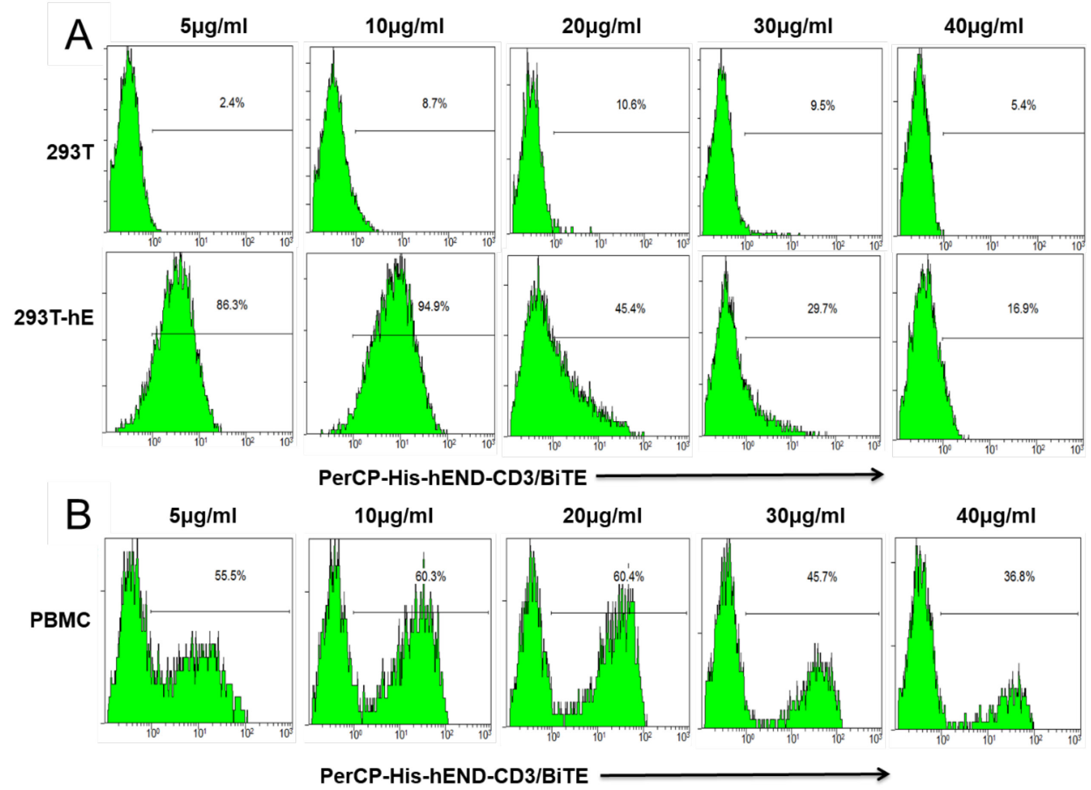

Supplemental Figure 2. Verification of the hEND-CD3/BiTE binding ability to target cells. (A) Concentration gradient of END-CD3/BiTE binding to endoglin. (B) Concentration gradient of END-CD3/BiTE binding to PBMC.

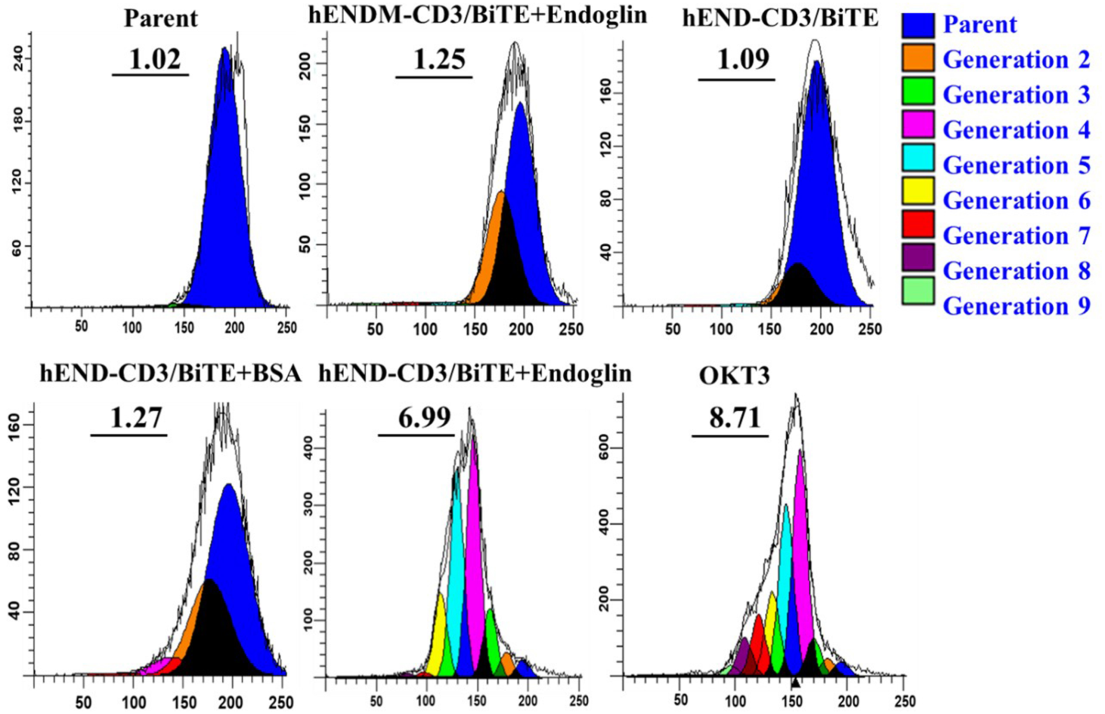

Supplemental Figure 3. hEND-CD3/BiTE induces human T lymphocyte proliferation index.

The mean proliferation index of T lymphocytes induced by hEND-CD3/BiTE alone was 1.07; by hEND-CD3/BiTE plus BSA was 1.27; by the control antibody hENDM-CD3/BiTE plus recombinant endoglin was 1.19; by hEND-CD3/BiTE plus recombinant endoglin was 6.10; by OKT3 in the positive control group was 7.69.

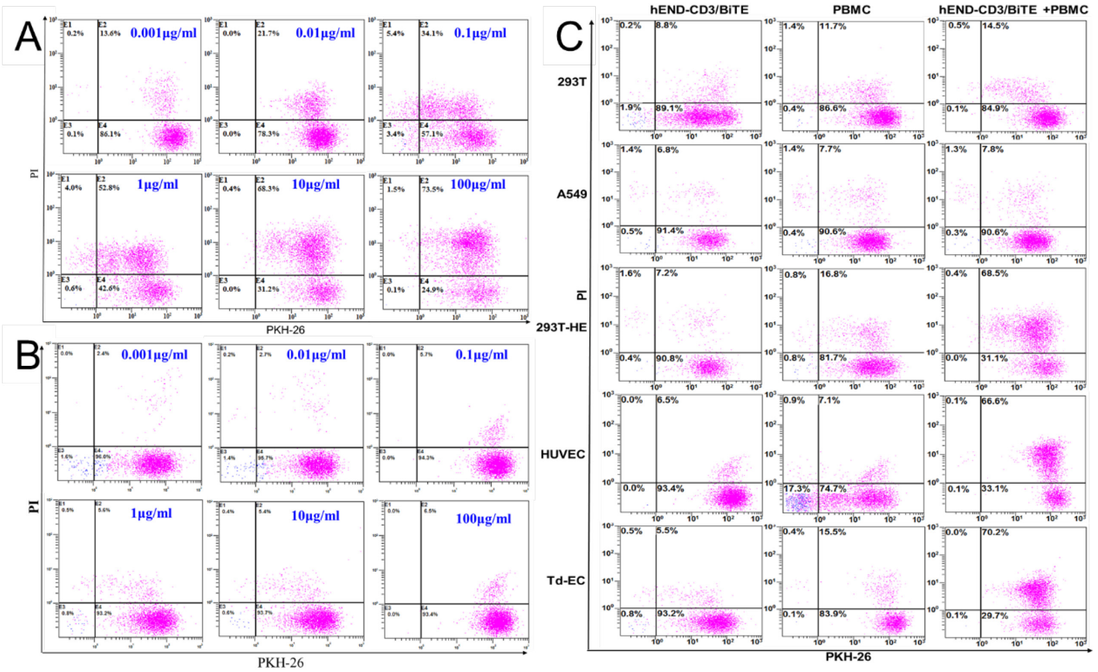

Supplemental Figure 4. Concentration gradient of cytotoxicity induced by hEND-CD3/BiTE in human T lymphocytes. A: 293T-HE; B: 293T. C: HEND-CD3/BiTE induced T cell cytotoxicity to 293T-HE, HUVEC and Td-EC cells expressing endoglin, with killing indexes of 66.9%, 61.22%, and 64.8% respectively, while the killing indexes of 293T and A549 cells expressing no endoglin were 9.9% and 2.4%, respectively.

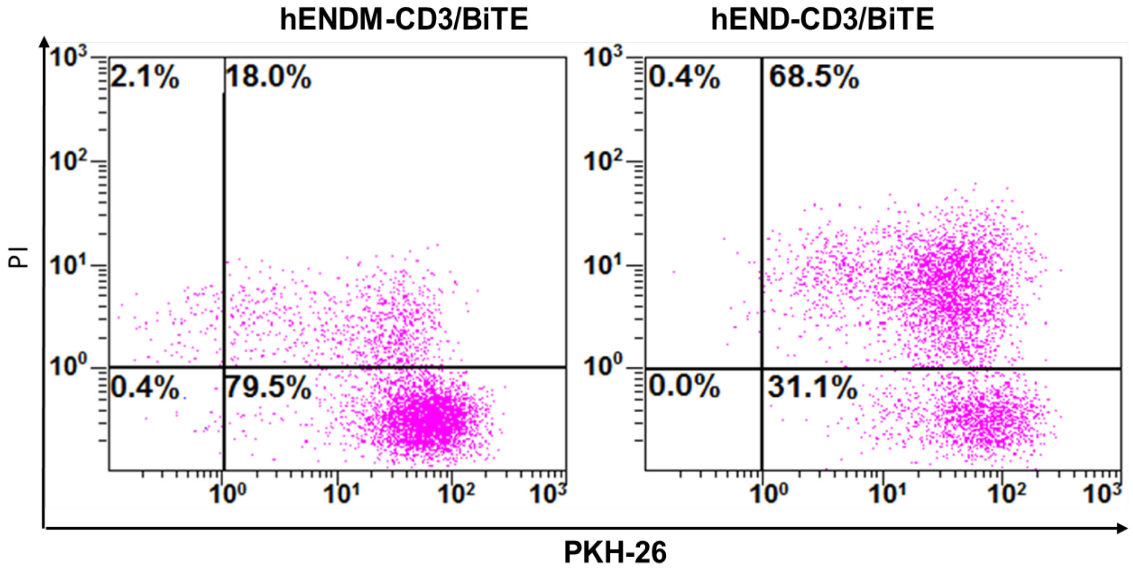

Supplemental Figure 5 hEND-CD3/BiTE induces T cell killing specificity. The killing index of hEND-CD3/BiTE for 293T-hE was significantly higher than that of the negative control hENDM-CD3/BiTE. *represent P<0.01

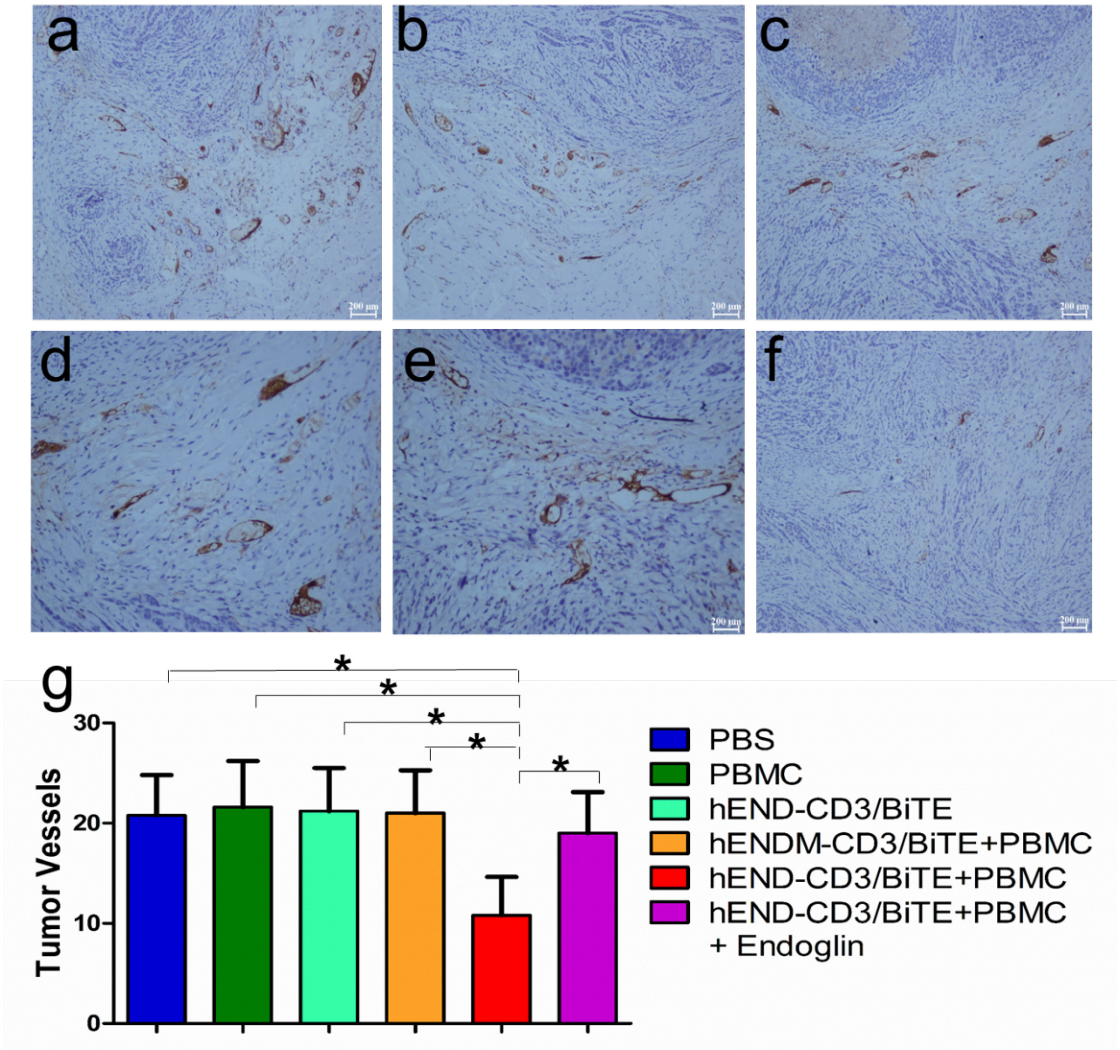

Supplemental Figure 6 hEND-CD3/BiTE together with PBMCs reduces tumor vascular density. The vascular endothelial cells within the tumors were detected via immunohistochemical staining of CD34; representative IHC images (200×) from each group. a. PBS; b. PBMC; c. hEND-CD3/BiTE; d. hENDM-CD3/BiTE+PBMC; e. hEND-CD3/BiTE+PBMC+Endoglin; f. hEND- CD3/BiTE +PBMC; g. $CD34^+$ vessels in the tumors were quantified and compared between groups.

Table 1 hEND-CD3/BiTE gene sequence

GAATTCATGCAGGTACAGCTGCAGCAGTCAGGCCCAGGACTGGTGAGGCCTTCGGAGACCCTATCTCTAATCTGCTCTGTCTCT
EcoRI
GGTGGCTCCATCACTAGTGATACTTACTACTGGGCCTGGCTCCGCCAGACCCCAGGGAAGAGGCTGGAGTGGATTGGGAGT
HCDR1
ATCTATTTTACTGGCAGAACCTTCTACAACCCGTCCCTCAAGACTGACTTCTGTGACCGCCGCAGACACGGCTCTTTATTATTGT
HCDR2
GCAAGAGACGGGGACGGTGCTATCTCTGGAGGCAACTGGTTCGACCCCTGGGGCCAGGGAACCCTGGTCACCGTCTCCTCA
HCDR3
GGCGGCGGCGGCTCTGGCGGAGGTGGCAGCGGCGGTGGCGGATCCCAGTCTGCCCTGACTCAGCCTGCCTCCGTGTCTGGGTCT
Connection sequence
CCTGGACAGTCGATCACCATCTCCTGCACTGGAACCAGCAGTGATGTTGGGAGTTATAACCTTGTCTCCTGGTACCAACAGCACCCA
LCDR1
GGCAAAGCCCCCAAACTCATGATTTATGAGGGCAGTAAGCGGCCCTCAGGGGTTTCTAATCGCTTCTCTGGCTCCAAGTCTGGC
LCDR2
AACACGGCCTCCCTGACAATCTCTGGGCTCCAGGCTGAGGACGAGGCTGATTATTACTGCTGCTCATATGCAGGTAGTAGCACTTAT
LCDR3
GTGGTATTCGGCGGAGGGACCAAGCTGACCGTCCTAGGTGGCGGCGGTGGCGGCAGCGGCGGAGGCGGAAGCGGAGGCGGAGGAAGC
Connection sequence
ATGCAGGTGCAGCTGTTGGAGTCTGGGGCTGAACTGGCAAGACCTGGGGCCTCAGTGAAGATGTCCTGCAAGGCTTCTGGCTACACC

TTTACTAGGTACACGATGCACTGGGTAAAACAGAGGCCTGGACAGGGTCTGGAATGGATTGGATACATTAATCCTAGCCGTGGTTAT

ACTAATTACAATCAGAAGTTCAAGGACAAGGCCACATTGACTACAGACAAATCCTCCAGCACAGCCTACATGCAACTGAGCAGCCTG

ACATCTGAGGACTCTGCAGTCTATTACTGTGCAAGATATTATGATGATCATTACTGCCTTGACTACTGGGGCCAAGGCACCCTGGTC

ACCGTCTCCTCAGGTGGCGGTGGCTCCGGCGGTGGTGGGTCGGGTGGCGGCGGATCTGACATTGTGATGACCCAGTCTCCAGCA
Connection sequence
ATCATGTCTGCATCTCCAGGGGAGAAGGTCACCATGACCTGCAGTGCCAGCTCAAGTGTAAGTTACATGAACTGGTACCAGCAGAAG

TCAGGCACCTCCCCCAAAAGATGGATTTATGACACATCCAAACTGGCTTCTGGAGTCCCTGCTCACTTCAGGGCCAGTGGGTCTGGG

ACCTCTTACTCTCTCACAATCAGCGGCATGGAGGCTGAAGATGCTGCCACTTATTACTGCCAGCAGTGGAGTAGTAACCCATTCACG

TTCGGCTCGGGGACCAAGCTGGAGATCAAACGTTAGCTCGAG
XhoI

Table 2 hEND-CD3/BiTE amino acid sequence

Met Gly Ser Ser His His His His His His Ser Ser Gly Leu Val Pro Arg
Gly Ser His Met Ala Ser Met Thr Gly Gly Gln Gln Met Gly Arg Gly Ser
Glu Phe Met Gln Val Gln Leu Gln Gln Ser Gly Pro Gly Leu Val Arg Pro
Ser Glu Thr Leu Ser Leu Ile Cys Ser Val Ser Gly Gly Ser Ile Thr Ser
Asp Thr Tyr Tyr Trp Ala Trp Leu Arg Gln Thr Pro Gly Lys Arg Leu Glu
Trp Ile Gly Ser Ile Tyr Phe Thr Gly Arg Thr Phe Tyr Asn Pro Ser Leu
Lys Ser Arg Leu Thr Ile Ser Leu Asp Thr Ser Lys Ser Gln Phe Ser Leu
Lys Leu Thr Ser Val Thr Ala Ala Asp Thr Ala Leu Tyr Tyr Cys Ala Arg
Asp Gly Asp Gly Ala Ile Ser Gly Gly Asn Trp Phe Asp Pro Trp Gly Gln
Gly Thr Leu Val Thr Val Ser Ser Gly Gly Gly Gly Ser Gly Gly Gly Gly
Ser Gly Gly Gly Gly Ser Gln Ser Ala Leu Thr Gln Pro Ala Ser Val Ser
Gly Ser Pro Gly Gln Ser Ile Thr Ile Ser Cys Thr Gly Thr Ser Ser Asp
Val Gly Ser Tyr Asn Leu Val Ser Trp Tyr Gln Gln His Pro Gly Lys Ala
Pro Lys Leu Met Ile Tyr Glu Gly Ser Lys Arg Pro Ser Gly Val Ser Asn
Arg Phe Ser Gly Ser Lys Ser Gly Asn Thr Ala Ser Leu Thr Ile Ser Gly
Leu Gln Ala Glu Asp Glu Ala Asp Tyr Tyr Cys Cys Ser Tyr Ala Gly Ser
Ser Thr Tyr Val Val Phe Gly Gly Gly Thr Lys Leu Thr Val Leu Gly Gly
Gly Gly Gly Gly Ser Gly Gly Gly Gly Ser Gly Gly Gly Gly Ser Met Gln
Val Gln Leu Leu Glu Ser Gly Ala Glu Leu Ala Arg Pro Gly Ala Ser Val
Lys Met Ser Cys Lys Ala Ser Gly Tyr Thr Phe Thr Arg Tyr Thr Met His
Trp Val Lys Gln Arg Pro Gly Gln Gly Leu Glu Trp Ile Gly Tyr Ile Asn
Pro Ser Arg Gly Tyr Thr Asn Tyr Asn Gln Lys Phe Lys Asp Lys Ala Thr
Leu Thr Thr Asp Lys Ser Ser Ser Thr Ala Tyr Met Gln Leu Ser Ser Leu
Thr Ser Glu Asp Ser Ala Val Tyr Tyr Cys Ala Arg Tyr Tyr Asp Asp His
Tyr Cys Leu Asp Tyr Trp Gly Gln Gly Thr Leu Val Thr Val Ser Ser Gly
Gly Gly Gly Ser Gly Gly Gly Gly Ser Gly Gly Gly Gly Ser Asp Ile Val
Met Thr Gln Ser Pro Ala Ile Met Ser Ala Ser Pro Gly Glu Lys Val Thr
Met Thr Cys Ser Ala Ser Ser Ser Val Ser Tyr Met Asn Trp Tyr Gln Gln
Lys Ser Gly Thr Ser Pro Lys Arg Trp Ile Tyr Asp Thr Ser Lys Leu Ala
Ser Gly Val Pro Ala His Phe Arg Ala Ser Gly Ser Gly Thr Ser Tyr Ser
Leu Thr Ile Ser Gly Met Glu Ala Glu Asp Ala Ala Thr Tyr Tyr Cys Gln
Gln Trp Ser Ser Asn Pro Phe Thr Phe Gly Ser Gly Thr Lys Leu Glu Ile
Lys Arg
